# Supplementary material for: Ectomycorrhizal Community Shifts at a Former Uranium Mining Site
Source: J Fungi (Basel). 2023 Apr 18;9(4):483. doi: 10.3390/jof9040483 (PMC10144560; doi:10.3390/jof9040483)
Supplement: Supplementary file 1 [file jof-09-00483-s001.zip › jof-2279534-supplementary.pdf]

Supplementary Materials

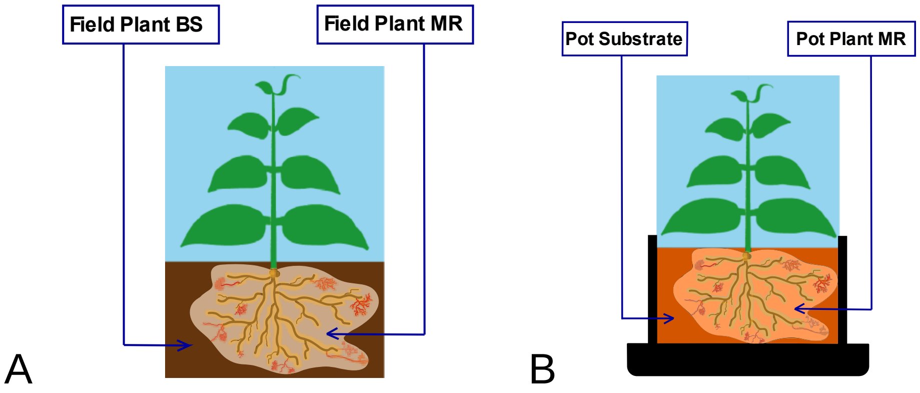

Figure S1. Schemes of soil sampling. A, at the test field; B, from pots. MR, mycorrhizosphere; BS, bulk soil.

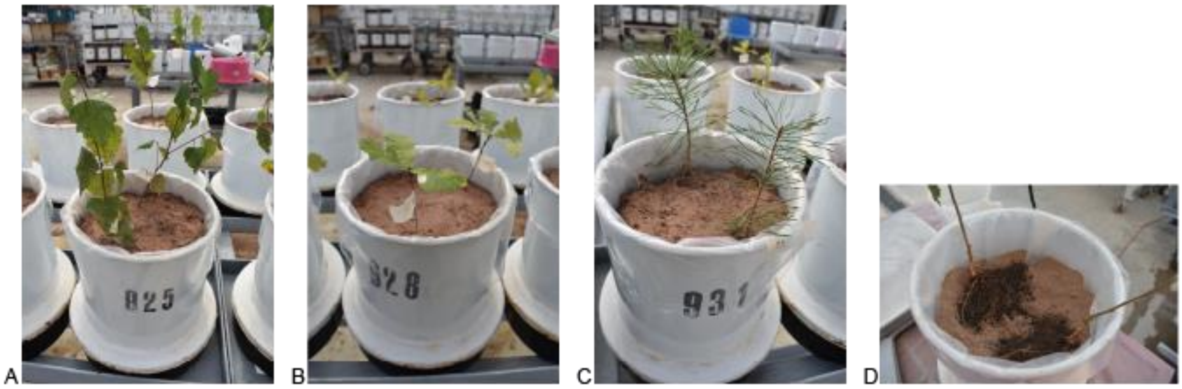

Figure S2. Images of pot variants. A, pot birches; B, pot oaks; C, pot pines; D, the process of inoculation of plants with the ectomycorrhizal fungi.

Table S1. Overview of pot experiment variants.

**Non-inoculated variants**				**Inoculated variants**			
control	birch	oak	pine	control	birch	oak	pine
3 pots	6 pots	6 pots	5 pots	3 pots	7 pots	7 pots	5 pots
-	12 plants	12 plants	10 plants	-	14 plants	14 plants	10 plants

Table S2. Characteristics of exploration types of mycorrhiza according to Agerer [13].

Exploration type	Hyphal mantle	Emanating hyphae	Rhizomorphs	Hydrophobicity	Contact with a substrate
Contact	Smooth	Rare	No	Hydrophilic	Close contact with a substrate via mantle
Short distance	Different types	Voluminous	No	Hydrophilic	Close contact with a substrate via mantle and emanating hyphae
Medium distance fringe subtype	Different types	Voluminous	Fans of ramified and repeatedly interconnected rhizomorphs	Hydrophilic	Extended contact with the soil through rhizomorphs
Medium distance mat subtype	Different types	Presented	Mats of densely packed undifferentiated rhizomorphs	Hydrophobic	Extended contact with the soil through rhizomorphs
Medium distance smooth subtype	Smooth	Rare	Rhizomorphs un- or slightly differentiated with a thick central hypha	Hydrophobic	Extended contact with the soil through rhizomorphs
Long-distance	Smooth	Presented	Rare but highly differentiated	Hydrophobic	Long distribution in the soil

Table S3. Morphological description of morphotypes.

Host	Variant	Morphotype	Morphology	Emanating hyphae	Rhizomorphs	Exploration type
Birch	Field plant	B_F_MT1	unramified/monopodial pinnate, straight unramified ends, smooth mantle, light brown to dark brown colour	lacking	no	contact
		B_F_MT2	unramified, straight unramified ends, woolly mantle, black colour	frequent, black	no	short
		B_F_MT3	unramified/monopodial pinnate, straight unramified ends, smooth mantle, beige colour with silver dots	lacking	no	contact
	Pot non-inoculated plant	B_non_inoc_MT1	unramified, straight unramified ends, smooth mantle, light brown colour	lacking	no	contact
		B_non_inoc_MT2	unramified, straight unramified ends, woolly mantle, black colour	frequent, black	no	short
		B_non_inoc_MT3	unramified, straight unramified ends, woolly mantle, light brown colour with silver dots	frequent, white	no	short
		B_non_inoc_MT4	unramified, straight unramified ends, woolly mantle, black colour, brown tips	frequent, black	no	short
		B_non_inoc_MT5	unramified, straight/bent unramified ends, grainy mantle, dark brown colour	infrequent, white/beige	no	short
	Pot inoculated plant	B_inoc_MT1	unramified, straight unramified ends, smooth mantle, light brown to beige colour	lacking	no	contact
		B_inoc_MT2	unramified, straight unramified ends, woolly mantle, black colour	frequent, black	no	short
		B_inoc_MT3	unramified/monopodial pinnate, straight unramified ends, smooth mantle, dark brown colour	lacking	no	contact
Oak	Field plant	O_F_MT1	unramified, straight unramified ends, woolly mantle, black colour	frequent, black	no	short
		O_F_MT2	unramified, straight unramified ends, smooth mantle, light brown colour	lacking	no	contact
		O_F_MT3	unramified, straight unramified end, grainy mantle, dark brown to black colour	infrequent, white	no	contact
		O_F_MT4	unramified/irregular pinnate/dichotomous-like, tortuous unramified ends, stringy mantle, brown colour	abundant, white	yes, with restricted points of connection with mantle and interconnected filaments, white colour	medium, fringe subtype
	Pot non-inoculated plant	O_non_inoc_MT1	unramified, straight unramified ends, woolly mantle, black colour	infrequent, black	no	short
		O_non_inoc_MT2	unramified, straight unramified ends, smooth mantle, light brown colour	lacking	no	contact
		O_non_inoc_MT3	unramified, straight unramified ends, woolly mantle, silver colour, light brown tips	infrequent, white	restricted points of connection with mantle, unsheathed, white colour	medium, smooth subtype
		O_inoc_MT1	unramified, straight unramified ends, smooth/grainy mantle, beige colour	lacking	no	contact
		O_inoc_MT2	unramified, straight unramified ends, woolly mantle, black colour	abundant, black	no	short

Host	Variant	Morphotype	Morphology	Emanating hyphae	Rhizomorphs	Exploration type
		O_inoc_MT3	unramified/dichotomous, straight unramified ends, woolly mantle, white to grey colour	infrequent, white	no	short
Pine	Field plant	P_F_MT1	unramified/dichotomous, straight unramified ends, reticulate mantle, light brown colour	lacking	no	contact
		P_F_MT2	unramified, straight unramified ends, woolly mantle, black colour	abundant, black	no	short
		P_F_MT3	coralloid, straight unramified ends, reticulate mantle, white colour	lacking	yes, restricted points of connection with mantle, hairy, white-pinkish colour	medium, fringe subtype
		P_F_MT4	dichotomous, straight unramified ends, woolly mantle, brown colour	abundant, white	yes, hyphal fans, interconnected filaments, white colour	medium, mat subtype
	Pot non-inoculated plant	P_non_inoc_MT1	unramified/dichotomous, straight unramified ends, smooth mantle, light brown to beige colour	lacking	no	contact
		P_non_inoc_MT2	unramified/dichotomous, straight unramified ends, smooth mantle, white to grey colour	lacking	no	contact
		P_non_inoc_MT3	unramified, straight unramified ends, woolly mantle, dark brown to black colour	abundant, black	no	short
		P_non_inoc_MT4	unramified/dichotomous, straight unramified ends, woolly/grainy mantle, light beige colour	frequent, white	yes, growing off in flat angles, interconnected filaments, yellow colour	long
	Pot inoculated plant	P_inoc_MT1	unramified/dichotomous, straight unramified ends, smooth mantle, light brown to beige colour	lacking	no	contact
		P_inoc_MT2	unramified, straight unramified ends, woolly mantle, black colour, brown tips	abundant, black	no	short
		P_inoc_MT3	unramified, straight unramified ends, woolly/grainy mantle, beige colour with silver dots	infrequent, white	no	short
		P_inoc_MT4	unramified, bent unramified ends, woolly mantle, silver colour	infrequent, white	no	short

>B_F_MT1-ITS-1
gCACGCcaGGGCGTGTCCTCTCACATAAAATCCATCTCACCCTTTTGTGCACCACCGCGTGGGCACCCTTCGGGATCGAAC
AGATCCAGGAGGGGGCTTGCGTTTTCACACAAACCCCCTTTTAAAAGTGTAGAATGACCCCACTTTTGCGATAACACGCAA
TCAATACAACTTTCAACAACGGATCTCTTGGCTCTCGCATCGATGAAGAACGCAGCGAAATGCGATACGTAATGTGAATTG
CAGAATTCAGTGAATCATCGAATCTTTGAACGCACCTTGCGCCCCTTGGTATTCCGAGGGGCACACCCGTTTGAGTGTCGT
GAAAATCTCAACCTTCTCGGTTTCTTCTGAACACCGAAGGAGGCTTGGACTTTGGAGGCCTTTGCTGGCGTCTCTCACCAG
CTCCTCTCAAACGAATTAGCGGGGTCCTCTTTGCCGATCCTCGACATGTGATAAGATGTTTCCATGTCTTGGCTTTATGGCT
CTGTTGCCTTTGGGACCCGCTTCTAACCGTCTCAGCGAGACAACGTTCGAGCGTGTCTCCCCTCTCGGGGAAACGCTCTT
GAACCCACGAAACCTTGACCTCAAATCGGGTGAGAcTACCCGCTGAACTTAAGCATATCAATAAGCGgAGGAaaa

>B_F_MT2-ITS-1
tGGgTTCtATTCCcAAACCGtGTATACATACCTTTGTTGCTTTGGCaGGCCGCCTCTTAgGCGTCGGCTCCGGCTGACTGCGC
CTGCCAGAGAACCCaAACTCGTTTGTTTAGTGATGTCTGAGTACTATATAATAGTTAAAACTTTCAACAACGGATCTCTTGGT
TCTGGCATCgATGAAGAACGCAGCGAAATGCGATAAGTAATGTGAATTGCAGAATTCAGTGAATCATCgAATCTTTGAACGC
ACATTGCGCCCCTTGGTATTCCGAGGGGCATGCCTGTTCGAGCGTCATTATAACCACTCAAgCCtCgGCTTGGTCTTGGGG
TTCGCGGTCTCGCGGCCCTTAAAATCAGTGGCGGTGCCGTCTGGCTCTAAgCGTAGTAATTCTCTCGCTATAGGGTCCCG
GCGGTCACCTGtCAgAACCCCCCCATTTTTTACGGtTGACCtCggAtCAGGtagGGataCCcgctgAacTtaAGCATATCAAtaaGcgGA
ggA

>B_F_MT3-ITS-1
tCaGGTTGTTGCTGGCTCCCTGAGCATGTGCACACTTGTCATCTTTACTTTTCCCCAATGTGCACATATTGTAGGCCTGGAG
CTCTTTTGATTATCTTGTATAGATTGAGGACTGCTGTGCTTCAGCCAGCTTTTCCTTGTGTTTCCAGGTCTATGTTTACCTTC
ACAAACTCTGAATATGTTTAGAATGTTGAAATATAATATATATATACAACTTTCAGCAACGGATCTCTTGGCTCTCGCATCGA
TGAAGAACGCAGCGAAATGCGATAAGTAATGTGAATTGCAGAATTCAGTGAATCATCGAATCTTTGAACGCATCTTGCGCT
CCTTGGTATTCCGAGGAGCATGCCTGTTTGAGTGTCATTAATCTCTCAACCACACCAATCTTTTTGGCTTGGTGTGGCTTTT
GGATGTGGGGGTTTTATTTTGCTGGCTCCTATTAAATGAGGTCAGCTCCCCTGAAACTTATTAGCGGTATCTGAGCAGAGA
GACCTACTACAGGTGTGATAAATTATCTATGCCTTGGTCATACTGCATGAACAGATTGTGCTGCTTCCAGTGAATCATTTGA
CAATTTTGACCTCAAATCAGGTAGGACTACCCGCTGAACTTAAGCATATCAATAAGCGGAGGAa

>O_F_MT1-ITS-1
aacGGGTTCTATTCCccaACCGTGTaTACATACCTTTGTTGCTTTGGCAGGCCGCCTTTTAGGCGTCGGCTTCGGCTGACTG
CGCCTGCCAGAGGACCCAAACTCGTTTGTTTAATGATGTCTGAGTACTATATAATAGTTAAAACTTTCAACAACGGATCTCT
TGGTTCTGGCATCgATGAAgAACGCAgCGAAATGCGATAAgTAATGTGAATTGCAaAATTCAGTGAATCATCgAATCTTTGAA
CgCaCATTGCgCCCCTTGGTATTCCGAGGGGCaTGcCTGTTCaAgcgtCATTATaACCACTCAAgCCTCgGCTTGGTCTTGgggtt
CgCGGtCTCgCgtcctTTAAAATCagtGGCGGTGCCGtCTGGcTCTAAgCgTAgtAATTCTCtCgcTATAGGGtCCCGGcGGTTGCCT
GCCagAACCCccCATTTTTTAcggtTGACCTCGgATcagGtAgGGATACcCGCTGAaCTTaagcatAtcAa

>O_F_MT3-ITS-1
GaatGGgtTCTATTCCcaAACCgTGTATACaTACCTTTGTTGCTTTGGCAGGCCGCCTTTTAGGCGTCGGCTCCGGCTGACTG
CGCCTGCCAGAGGACCCAAACTCGTTTGTTTAGTGATGTCTGAGTACTATATAATAGTTAAAACTTTCAACAACGGATCTCT
TGGTTCTGGCATCGATGAAGAACGCAgCGAAATGCGATAAGTAATGTGAATTGCAgAATTCAGTGAATCATCgAATCTTTGA
ACGCACATTGCGCCCCTTGGTATTCCGAGGGGCATGCCTGTTCaAgCGTCATTATAACCACTCAAgCCTCgGCTTGgTCTTG
gggttCGCGGTCCCgCGGCCCTTAaAATCagTGGCGGTGCCgtCTGGcTCTAAgCgtActAaTTCtctcgctATGGGGtCcCGGCGGtTg
cctGccagAACcccccatTTtttacgg

>O_F_TM4-ITS-1
tGatGGgTTGtTgcTGGTTCTCTAGGGAGCATGTGCACGCCTTGTCATCTTTATATCTCCACCTGTGCACCTTTTGTAGACCTT
CCAGGTCTATGTTGCTTCATTTACCCCAATGTATGTTAATAGAATGTTGTGCCTACAAAATACATACAACTTTCAGCAACGGA
TCTCTTGGCTCTCGCATCGATGAAGAACGCAGCGAAATGCGATAAGTAATGTGAATTGCAgAATTCAgTGAATCATCGAATC
TTTGAACGCACCTTGCGCTCCTTGGTATTccgAgGAGCATGCCTGTTTGAgTGTCATTAATATATATCAACCTCTCTCTTGTTG
GAGtGgtttGgATgtgggggttTtgTatgtaTTTTGTAaGcACAACATTCtattAacaTACaTTGGGGTaaaTGaagcaaCATAgAcctGGAAgGTC
tacaAaAgGTGCACAGGTggAgatataaAGATgAcaagGcgTGcacaTg

>P_F_MT1-ITS-1
aTggaaAaaCGGGGTTGTTGCTGGCCTCCAatgGGGGCACGTGCACGcTCTGTTTACACATCCACTCACACCTGTGCACCCTC
TGTAGTCCTATGGTTCGGAAGACCCCGTCTTCCTTCTGTAGCTCTACGTCTTTACACACACACTGTAGCGATGTCTCATGGA
ATGTTTTTATGCGTTTAACGCGATACAATACAACTTTCAGCAACGGATCTCTTGGCTCTCGCATCGATGAAGAACGCAGCGA
AATGCGATAAGTAATGTGAATTGCAGAATTCAGTGAATCATCGAATCTTTGAACGCACCTTGCGCCCCTTGGTTATTCCTTG
GAGCATGCCTGTTTGAGTATCATGAACACCTCAACTCTCATGGTCTTCCATGATGAGCTTGGACTTTGGGGGTTTGCTGGC
CCTGCGGCCGGCTCCTCTCAAATGAATCAGCTTGCCAGTGTTTGGTGGCATCATGGGTGTGATAACTATCTACGCTCGCG
GTCgTCTGCCAGGTAACCCTCAGCGATGAGGGTTCATTGGAGCTCATAAACGTCTCTCCTCGGCGAGGACAACTTTTGAAC
GTTCGATCTCAAATCAGGTAGGATTACCCGCTGAACTTAAGCATATCAATAAGCGGAGGAA

>P_F_MT2-ITS-1
aacGGGTTCTATTCCcaaACCGTGTaTACATACCTTTGTTGCTTTGGCAGGCCGCCTTTTAGGCGTCGGCTTCGGCTGACTG
CGCCTGCCAGAGGACCCAAACTCGTTTGTTTAATGATGTCTGAGTACTATATAATAGTTAAAACTTTCAACAACGGATCTCT
TGGTTCTGGCATCgATGAAgAACGCAgCGAAATGCGATAAgTAATGTGAATTGCAaAATTCAGTGAATCATCgAATCTTTGAA
CgCaCATTGCgCCCCTTGGTATTCCGAGGGGCaTGcCTGTTCaAgcgtCATTATaACCACTCAAgCCTCgGCTTGGTCTTGgggtt
CgCGGtCTCgCgtcctTTAAAATCagtGGCGGTGCCGtCTGGcTCTAAgCgTAgtAATTCTCtCgcTATAGGGtCCCGGcGGTTGCCT
GCCagAACCCccCATTTTTTAcggtTGACCTCGgATcagGtAgGGATACcCGCTGAaCTTaagcatAtcAa

>P_F_MT3-ITS-1
aaaaaaTTCGGAaGGGGTTGTAGCTGGCCGAGGAAACGAGGCATGTGCACGCTCTTCTGTTTTTCATAACTCACCTGTGCAC
CTAATGTAgGATGCTCCTCTTTCGGGAGGGGGGACCTATGTCTTTGTATAACTCTTCGTGTAGAAAGTCTTAGAATGTTTAC
TATCAGAGAGTCGCGACTTCTAGGAGACGCGAATCTTTGAGATAAAAGTTATTACAACTTTCAGCAATGGATCTCTTGGCTC
TCGCATCGATGAAGAACGCAGCGAAAAGCGATATGTAATGTGAATTGCAGATCTACAGTGAATCATCGAATCTTTGAACGC
ACCTTGCGCTCCTCGGTGTTCCGAGGAGCATGCCTGTTTGAGTGTCAGTAAATTCTCAACCCCTCTTGATTTGTTTCGAGG
GGGAGCTTGGATTGTGGGGGCTGCCGGAGACTAGGACTCGTCCTTGACTCGGGCTCTCCTTAAATGCATCGGCTTGCGG
TCGACTTTCGACTTTGCGCGACAAGGCTTTCGGCGTGATAATGATCGCCGTTCGCTGAAGCGCATGAATGAAGGTTCCGT
GCCTCTAATTCGTCGACTTAGTATCTCTTCCGAGAGAAAACGTCTTCTTCATTGACTTTTGACCTCAAATCAGGTAGGACTA
CCCGCTGAACTTAAGCATATCAATAAGCGGaggAaGaacaaTtAACgAAGATtatTCGGAGGGGTTGTAGCTGGCCGAGGAAAcg
aaGGCaggtGccgcTCTTCTGTTTTTCaaaactCACTGtGCACTAAAGtAGGagGCTCtCTTTCGggaGGggGGAccTATGTcTTTGTAT
AACTCTtctgTAaaagGtcTTAGA

>P_F_MT4-ITS-1
CTGGTTGGGTTGTTGCTGGCTCTCCGGGGCATGTGCACACCTGACACCAACTTTTTACCACCTGTGCACCTTCTGTAGACC
TGGATAAACTCTCGAGGAAACTCGGTTTGAGGACTGCTGTGTGTAAAAGCCAGCTTTCCTTaTAACTTCCGGTCTATGTCTT
TATATATACCCCTACAGTATGTCTAAGAATGTTTGTTGTTGGGCTTCAATGCCTTTAAACTTAATACAACTTTCAACAACGGA
TCTCTTGGCTCTCGCATCGATGAAGAACGCAGCGAAATGCGATAAGTAATGTGAATTGCAGAATTCAGTGAATCATCGAAT
CTTTGAACGCACCTTGCGCTCCTTGGTATTCCGAGGAGCATGCCTGTTTGAGTGTCATGAAATTCTCAACCTTTTCAGCTTT
CTTTGTTGATCAGGCTTGGATGTGGGAGTATGCGGGCTTCTCAGAAGTCGGCTCTCCTGAAATGCATTAGCGGGACCTTTG
TTGCCTGGCTTTGGTGTGATAATTATCTACGCCATTGTAGTCAAGCAGCTTaaAATGAGGTTTCagcTTCTAACcGTCTCCCCT
GAGACAACTTTtTGACAATTTGACCTCAAATCAGGTAGGACTACCCGCTGAACTTaaacATATcaataag

>O_inoc_MT3-ITS-1
GGGGatCGGAgAAatCCGTTCCTTCgaAGCCCTTTTaCCTCTTTCaCACCTGtGtGcACCCACGTTCgCAAGGtCcTTCgGGACCT
GCgCaTTCTATCTCgAAcTCgcAtGtctaCagAATGTCGCTagcATTTGAaATGAaaTAcAACTTTcatCAaTGGATCTCTTGgctCtCgC
ATCGATGAAGAAcgcagCGAAtCgcGATAAGTAATGtgaatTGcaGAttTTcCGTGAATCATCgAATCTTTGAACGCaCCTTGcgCTC
CTTGgTatTCCGAGGAGCATGcctGtttGAgTGtcaTCgAAATCTCaaACTGAgcCTTTTTGAcTTTGGTCaAAGGctTGgGTTTTGgAc
CGtTGGGAGTCTGCGGGTGACCCTCTCCGGGAcGtCgGCTCTCCTGAaATGcaTTAgcAgTGGgcaTGtGcaAgCCTTGcatGgcac
CAgCCTCtCCgAcgtcatAATGAtcgtCGTGggctGtaagCTGCaaaGGAtGTacCCCATGCTcCcTAAAcCTGcgAgcCCTCCCAGGcTCt
GCTTTcgAAGGcttGaCCTCaAATCAgGTAGGACTACCCGCTGAaCTTaagCATATCAaTA

>P_inoc_MT1-ITS-1
aaaCTTGAtcaGGTTGTTGCTGGCTCCCCTGAGCATGTGCACACTTGTCATCTTTACTTTTCCCCAATGTGCACATATTGTAG
GCCTGGAGCTCTTTTGATTATCTTGTATAGATTGAGGACTGCTGTGCTTCAGCCAGCTTTTCCTTGTGTTTCCAGGTCTATG
TTTACCTTCACAAACTCTGAATATGTTTAGAATGTTGAAATATAATATATATATACAACTTTCAGCAACGGATCTCTTGGCTCT
CGCATCGATGAAGAACGCAGCGAAATGCGATAAGTAATGTGAATTGCAGAATTCAGTGAATCATCGAATCTTTGAACGCAT
CTTGCGCTCCTTGGTATTCCGAGGAGCATGCCTGTTTGAGTGTCATTAATCTCTCAACCACACCAATCTTTTTGGCTTGGTG
TGGCTTTTGGATGTGGGGGTTTTATTTTGCTGGCTCCTATTAAATGAGGTCAGCTCCCCTGAAACTTATTAGCGGTATCTGA
GCAGAGAGACCTACTACAGGTGTGATAAATTATCTATGCCTTGGTCATACTGCATGAACAGATTGTGCTGCTTCCAGTGAAT
CATTTGACAATTTTGACCTCacaTCAgGTatgACTACCCGCTGAACTTaagcATATCAAtAAtc

>P_inoc_MT2-ITS-1
cCCAaacTcttttAtTagTGAtGtctGAgTaCTATATAAtAcTtAaaActTtcaACAAcggAtCTCTTGgtTCTGGcaTcgatgAacaacgcagcgaAatG
caAtaAgTAATGtgAATTGCatAatTcagTGAATCaTCaAATCTTTgAAcgcacaTTGCGCCcgcTggtaTTCCgGCGGgcATGCcTGtccgA
gcGtCattatGAccaAtcaagctctGcTTGgcCTTGgGGcCtGCTataCCGgCgGTCCTTaAaAtCagtGgcggtGCCgtCtGgCTCTAatcgtAtt

>P_inoc_MT3-ITS-1
cgGGTTCTATtCccaaaCCGTGTATACaTACCTTTGTTGCTTTGGcagGcCGCCTTTTAgGCGTCGGCTTCGGCTGACTGCGCCT
GCCAGAgGACCCaaAcTCgtTTGTTTAATGATGTCTGAgTACTATATAATAgTTAAAACTTTCAACAACGGATCTCTTGGTTCTG
GCATCGATGAAGAACGCAgCGAAATGCGATAAgTAATGTGAATTGCAGAATTCAGTGAATCATCGAATCTTTGAACGCACAT
TGCGCCCCTTGGTATTCCGAGGGGCATGcCtGtTCGAGCGTCATTATAACCACTCAAGCCTCGGCTTGGTCTTGGGGTTCgC
GgtCTCgCGTCctTTaAAAtCagtGGCGGTGCCGTCTGGCTCTAAgCGTAgtAATTCTCTCgCTATAGGGTCCCGGCGGTTGCCT
GCCAgAACCCCCCATTTTTTACGGTTGACCTCGGATCAgGTAGGGATACCCgCTgAAcTTAAgCatAtCAATAAgcggAgG

>P_inoc_MT4-ITS-1
aAAaaaaaaTTCGGAGGGGTTGTAGCTGGCCGAGGAAACGAGGCATGTGCACGCTCTTCTGTTTTTCATAACTCACCTGTGC
ACCTAATGTAGGATGCTCCTCTTTCGGGAGGGGGGACCTATGTCTTTGTATAACTCTTCGTGTAGAAAGTCTTAGAATGTTT
ACTATCAGAGAGTCGCGACTTCTAGGAGACGCGAATCTTTGAGATAAAAGTTATTACAACTTTCAGCAATGGATCTCTTGGC
TCTCGCATCGATGAAGAACGCAGCGAAAAGCGATATGTAATGTGAATTGCAGATCTACAGTGAATCATCGAATCTTTGAAC
GCACCTTGCGCTCCTCGGTGTTCCGAGGAGCATGCCTGTTTGAGTGTCAGTAAATTCTCAACCCCTCTTGATTTGTTTCGA
GGGGGAGCTTGGATTGTGGGGGCTGCCGGAGACTAGGACTCGTCCTTGACTCGGGCTCTCCTTAAATGCATCGGCTTGC
GGTCGACTTTCGACTTTGCGCGACAAGGCTTTCGGCGTGATAATGATCGCCGTTCGCTGAAGCGCATGAATGAAGGTTCC
GTGCCTCTAATTCGTCGACTTAGTATCTCTTCCGAGAGAAAACGTCTTCTTCATTGACTTTTGACCTCAAATCAGGTAGGAC
TACCCGCTGAACTTAAGCATATCAATAAggcGGAAGGAaaaAaggg

>O_non_inoc_MT3-ITS-1
CCGCTGCTTGCaGCGGGCTGCGAAGGGAGAtATTATACACCCTGTACTTTGTTTACCTTTGTTGCTTTGGCGGGCCGTCCG
TTTAGGCGTTGGCTCCGGCCGATCGTGCCCGCCAGAGGACCCAAACTCTTTTATTAGTGATGTCTGAGTACTATATAATAG
TTAAAACTTTCAACAACGGATCTCTTGGTTCTGGCATCGATGAAGAACGCAGCGAAATGCGATAAGTAATGTGAATTGCAGA
ATTCAGTGAATCATCGAATCTTTGAACGCACATTGCGCCCGCTGGTATTCCGGCGGGCATGCCTGTCCGAGCGTCATTATG
ACCAATCAAGCTCTGCTTGGCCTTGGGGCCTGCTATACCGGCGGTCCTTAAAATCAGTGGCGGTGCCGTCTGGCTCTAAG
CGTAGTAATTCTCCTCGCTATAGAGCTCCAGACGTCCACCCGCCAGAACCCCCAACTTTCTCAAGGTTGACCTCGGATCAG
GTAGGGATACCCGCTGAACTTAAGCATATCAATAAGCGGAGGAA

>P_non_inoc_MT1-ITS-1
aCTTGATcaGGTTGTTGCTGGCTCCCCTGAGCATGTGCACACTTGTCATCTTTACTTTTCCCCAATGTGCACATATTGTAGGC
CTGGAGCTCTTTTGATTATCTTGTATAGATTGAGGACTGCTGTGCTTCAGCCAGCTTTTCCTTGTGTTTCCAGGTCTATGTTT
ACCTTCACAAACTCTGAATATGTTTAGAATGTTGAAATATAATATATATATACAACTTTCAGCAACGGATCTCTTGGCTCTCG
CATCGATGAAGAACGCAGCGAAATGCGATAAGTAATGTGAATTGCAGAATTCAGTGAATCATCGAATCTTTGAACGCATCTT
GCGCTCCTTGGTATTCCGAGGAGCATGCCTGTTTGAGTGTCATTAATCTCTCAACCACACCAATCTTTTTGGCTTGGTGTG
GCTTTTGGATGTGGGGGTTTTATTTTGCTGGCTCCTATTAAATGAGGTCAGCTCCCCTGAAACTTATTAGCGGTATCTGAGC
AGAGAGACCTACTACAGGTGTGATAAATTATCTATGCCTTGGTCATACTGCATGAACAGATTGTGCTGCTTCCAGTGAATCA
TTTGACAATTTTGACCTCAAATCAGGTAGGACTACCCGCTGAACTTAAGCATATCAATAAGCGGAGGAa

Figure S3. ITS sequences for mycorrhizae fungus identification.

Table S4. ECM fungus identification by sequencing. For morphotypes with grey cells sequence analysis was not successful.

Morphotype	Length (bp)	Closest match, UNITE (parameters)				Closest match, NCBI (parameters)				
		Acc. #	UNITE name	Score (bits)	Identity %	Acc. #	Name	Query cover, %	Total score	Identity %
B_F_MT1	642	UDB011464	*Lactarius mammosus*	1104	100	KJ705202	*Lactarius mammosus*	97	1164	100
B_F_MT2	502	FN669230	*Meliniomyces bicolor*	883	99	FN669230	*Meliniomyces* sp.	100	900	99
B_F_MT3	638	UDB025177	*Inocybe lacera*	1088	100	HQ604443	*Inocybe lacera*	100	1157	99.4
B_non_inoc_MT1										
B_non_inoc_MT2										
B_non_inoc_MT3										
B_non_inoc_MT4										
B_non_inoc_MT5										
B_inoc_MT1										
B_inoc_MT2										
B_inoc_MT3										
O_F_MT1	492	UDB002586	*Meliniomyces bicolor*	874	99	KU176262	Uncultured *Meliniomyces*	98	865	99
O_F_MT2										
O_F_MT3	446	UDB026053	*Meliniomyces*	735	99	KY684476	Uncultured *Meliniomyces*	99	800	99.1
O_F_MT4	479	UDB0799592	*Cortinarius bivelus*	613	100	MK234571	*Cortinarius bivelus*	100	883	100
O_non_inoc_MT1										
O_non_inoc_MT2										
O_non_inoc_MT3	530	UDB028259	*Meliniomyces*	760	97	KT334710	Uncultured fungus	100	979	100
O_inoc_MT1										
O_inoc_MT2										
O_inoc_MT3	595	UDB07672649	*Pisolithus arhizus*	1018	100	FR748134	*Pisolithus capsulifer*	100	1094	99.8
P_F_MT1	632	UDB016490	*Tomentella ellisii*	1066	100	MZ773239	*Tomentella ellisii*	100	136	100
P_F_MT2	492	FN679031	*Meliniomyces bicolor*	854	99	KF428297	*Helotiaceae sp.*	100	876	98.8
P_F_MT3	848	UDB027685	*Rhizopogon mohelnensis*	1223	99	JX898967	Uncultured *Rhizopogon*	99	1549	99.7
P_F_MT4	644	UDB019593	*Tricholoma argyraceum*	1157	100	MG367247	*Tricholoma argyraceum*	100	1179	99.7
P_non_inoc_MT1	645	UDB025177	*Inocybe lacera*	1103	100	AB669659	Uncultured mycorrhizal fungus	100	1177	99.5
P_non_inoc_MT2										
P_non_inoc_MT3										
P_non_inoc_MT4										

Morphotype	Length (bp)	Closest match, UNITE (parameters)				Closest match, NCBI (parameters)				
		Acc. #	UNITE name	Score (bits)	Identity %	Acc. #	Name	Query cover, %	Total score	Identity %
P_inoc_MT1	640	UDB025177	*Inocybe lacera*	1081	100	FM992932	Uncultured ectomycor-rhiza (*Inocybe*)	98	1158	99.7
P_inoc_MT2	291	UDB028259	*Meliniomyces*	441	97	AB986370	*Hyaloscyphaceae* sp.	99	501	97.9
P_inoc_MT3	500	UDB017362	*Meliniomyces bicolor*	897	99	JX507662	*Helotiales* sp.	99	905	99.4
P_inoc_MT4	695	UDB025458	*Rhizopogon mohelnensis*	1216	99	HG426016	Uncultured *Rhizopogon*	97	1242	99.7

Table S5. One-Way ANOVA, Kruskal-Wallis and post hoc tests for significance of differences between variants of the experiment in diversity indices of ECM communities.

	ANOVA						Post Hoc comparison					
		Sum of Squares	df	Mean Square	F	p			Mean	SE	t	p
Simpson dominance index	variant	0.053	2	0.027	0.940	0.441	field birch	pot birch inoc	-0.067	0.146	-0.464	0.890
	Residuals	0.169	6	0.028				pot birch non-inoc	0.131	0.128	1.020	0.593
							pot birch inoc	pot birch non-inoc	0.198	0.153	1.293	0.449
	variant	0.120	2	0.060	2.245	0.176	field oak	pot oak inoc	0.153	0.119	1.286	0.446
	Residuals	0.187	7	0.027				pot oak non-inoc	0.275	0.137	2.013	0.179
							pot oak inoc	pot oak non-inoc	0.122	0.149	0.816	0.706
	variant	0.013	2	0.006	0.340	0.722	field pine	pot pine inoc	0.065	0.099	0.657	0.794
	Residuals	0.148	8	0.019				pot pine non-inoc	-0.021	0.099	-0.215	0.975
							pot pine inoc	pot pine non-inoc	-0.087	0.111	-0.780	0.725
Gini-Simpson index	variant	0.053	2	0.027	0.940	0.441	field birch	pot birch inoc	0.068	0.146	0.464	0.890
	Residuals	0.169	6	0.028				pot birch non-inoc	-0.131	0.128	-1.020	0.593
							pot birch inoc	pot birch non-inoc	-0.198	0.153	-1.293	0.449
	variant	0.120	2	0.060	2.245	0.176	field oak	pot oak inoc	-0.153	0.119	-1.286	0.446
	Residuals	0.187	7	0.027				pot oak non-inoc	-0.275	0.137	-2.013	0.179
							pot oak inoc	pot oak non-inoc	-0.122	0.149	-0.816	0.706
	variant	0.013	2	0.006	0.340	0.722	field pine	pot pine inoc	-0.065	0.099	-0.657	0.794
	Residuals	0.148	8	0.019				pot pine non-inoc	0.021	0.099	0.215	0.975
							pot pine inoc	pot pine non-inoc	0.087	0.111	0.780	0.725

Shannon diversity index	variant	0.168	2	0.084	1.208	0.362	field birch	pot birch inoc	0.082	0.228	0.362	0.931
	Residuals	0.417	6	0.069				pot birch non-inoc	-0.254	0.201	-1.263	0.463
							pot birch inoc	pot birch non-inoc	-0.337	0.241	-1.400	0.399
	Variant	0.340	2	0.170	2.650	0.139	field oak	pot oak inoc	-0.227	0.185	-1.226	0.476
	Residuals	0.449	7	0.064				pot oak non-inoc	-0.475	0.212	-2.242	0.131
							pot oak inoc	pot oak non-inoc	-0.248	0.231	-1.074	0.558
	Variant	0.025	2	0.013	0.271	0.770	field pine	pot pine inoc	-0.114	0.158	-0.721	0.758
	Residuals	0.375	8	0.047				pot pine non-inoc	-0.064	0.158	-0.405	0.915
							pot pine inoc	pot pine non-inoc	0.050	0.177	0.283	0.957
Berger-Parker index	Variant	0.036	2	0.018	0.683	0.540	field birch	pot birch inoc	-0.027	0.140	-0.197	0.979
	Residuals	0.157	6	0.026				pot birch non-inoc	0.122	0.123	0.993	0.608
							pot birch inoc	pot birch non-inoc	0.150	0.148	1.017	0.594
	Variant	0.086	2	0.043	1.278	0.337	field oak	pot oak inoc	0.131	0.134	0.978	0.612
	Residuals	0.237	7	0.034				pot oak non-inoc	0.233	0.154	1.515	0.341
							pot oak inoc	pot oak non-inoc	0.102	0.168	0.606	0.822
	Variant	0.023	2	0.012	0.476	0.638	field pine	pot pine inoc	0.086	0.115	0.750	0.742
	Residuals	0.197	8	0.025				pot pine non-inoc	-0.034	0.115	-0.297	0.953
							pot pine inoc	pot pine non-inoc	-0.120	0.128	-0.936	0.634

Asterisks represent significant p values (< 0.05). SE: standard error.

Table S6. Indices of similarity between ECM communities determined for trees within variants of the experiment.

Index	Estimate	Standard error	95%Lower	95%Upper
field birches				
Sorensen	0.970	0.017	0.936	1.000
Jaccard	0.889	0.052	0.786	0.991
Bray-Curtis	1.000	0.000	1.000	1.000
non-inoculated pot birches				
Sorensen	0.875	0.050	0.777	0.973
Jaccard	0.700	0.087	0.529	0.871
Bray-Curtis	0.930	0.057	0.819	1.000
inoculated pot birches				
Sorensen	0.924	0.151	0.628	1.220
Jaccard	0.858	0.214	0.440	1.277
Bray-Curtis	1.000	0.162	0.682	1.000
field oaks				
Sorensen	0.833	0.035	0.765	0.902
Jaccard	0.500	0.077	0.350	0.650
Bray-Curtis	0.907	0.047	0.816	0.999
non-inoculated pot oaks				
Sorensen	1.000	0.000	1.000	1.000
Jaccard	1.000	0.000	1.000	1.000
Bray-Curtis	1.000	0.000	1.000	1.000
inoculated pot oaks				

Sorensen	0.938	0.008	0.922	0.953
Jaccard	0.833	0.017	0.800	0.866
Bray-Curtis	1.000	0.005	0.990	1.000
field pines				
Sorensen	0.833	0.000	0.833	0.833
Jaccard	0.500	0.000	0.500	0.500
Bray-Curtis	0.954	0.011	0.932	0.975
non-inoculated pot pines				
Sorensen	0.955	0.017	0.922	0.987
Jaccard	0.875	0.039	0.798	0.952
Bray-Curtis	1.000	0.030	0.942	1.000
inoculated pot pines				
Sorensen	0.833	0.042	0.752	0.915
Jaccard	0.625	0.078	0.473	0.777
Bray-Curtis	0.852	0.074	0.706	0.997

Table S7. Kruskal-Wallis test and post hoc test output for significance of differences between variants of the experiment in relative abundance of exploration types of mycorrhiza.

Explora-tion type	Kruskal-Wallis			Post Hoc comparisons					
	Statistic	df	p	Comparisons		Mean	SE	t	p bonf
Contact	31.095	2	1.770e -7	birch field	birch pot inoc	-31.899	5.391	-5.917	**7.581e-8***
					birch pot non-inoc	-13.786	4.636	-2.974	**0.010***
				birch pot inoc	birch pot non-inoc	18.113	4.767	3.800	**6.523e-4***
	37.596	2	6.856e -9	oak field	oak pot inoc	-18.687	5.117	-3.652	**0.001***
					oak pot non-inoc	-20.625	5.532	-3.728	**9.337e-4***
				oak pot inoc	oak pot non-inoc	-1.938	5.117	-0.379	1.000
	8.054	2	0.018	pine field	pine pot inoc	-0.357	3.107	-0.115	1.000
					pine pot non-inoc	-6.935	2.892	-2.398	0.054
				pine pot inoc	pine pot non-inoc	-6.577	2.595	-2.535	**0.038***
Short distance	39.906	2	2.161e -9	birch field	birch pot inoc	4.934	3.604	1.369	0.520
					birch pot non-inoc	-15.585	3.099	-5.029	**4.594e-6***
				birch pot inoc	birch pot non-inoc	-20.519	3.187	-6.439	**5.738e-9***
	4.468	2	0.107	oak field	oak pot inoc	-7.559	3.422	-2.209	0.088
					oak pot non-inoc	-1.409	3.699	-0.381	1.000
				oak pot inoc	oak pot non-inoc	6.150	3.422	1.797	0.225
	48.719	2	2.635e -11	pine field	pine pot inoc	-0.485	3.776	-0.128	1.000
					pine pot non-inoc	16.468	3.515	4.685	**2.267e-5***
				pine pot inoc	pine pot non-inoc	16.952	3.153	5.376	**1.167e-6***
Medium distance	55.259	2	1.001e -12	oak field	oak pot inoc	2.537	2.760	0.919	1.000
					oak pot non-inoc	-12.325	2.984	-4.131	**2.171e-4***
				oak pot inoc	oak pot non-inoc	-14.863	2.760	-5.385	**1.315e-6***

Exploration type	Kruskal-Wallis			Post Hoc comparisons					
	Statistic	df	p	Comparisons		Mean	SE	t	p bonf
	10.619	2	0.005	pine field	pine pot inoc	0.459	0.166	2.763	**0.020***
					pine pot non-inoc	0.459	0.155	2.969	**0.011***
				pine pot inoc	pine pot non-inoc	7.772e-16	0.139	5.600e-15	1.000
Long distance	43.881	2	2.961e -10	pine field	pine pot inoc	0.996	4.116	0.242	1.000
					pine pot non-inoc	-19.863	3.831	-5.184	**2.727e-6***
				pine pot inoc	pine pot non-inoc	-20.859	3.437	-6.069	**4.811e-8***

Asterisks represent significant p values (< 0.05). SE: standard error.

Table S8. Correlation coefficients between soil characteristics and exploration types of mycorrhizae.

Soil characteristics	Contact exploration type	Short-distance exploration type	Medium-distance exploration type	Long-distance exploration type
Al	0.27	-0.33	0.03	0.12
Co	0.00	-0.07	-0.17	0.29
Cu	0.12	-0.08	-0.38	0.20
Fe	0.35	-0.13	-0.30	-0.17
Mn	-0.33	0.31	-0.08	0.08
Ni	-0.36	0.38	-0.10	-0.03
Pb	-0.31	0.20	0.06	0.10
Sr	-0.36	0.35	-0.08	-0.02
Zn	0.03	-0.29	0.07	0.35
Cs	0.01	-0.25	**0.40***	-0.03
U	-0.09	0.26	-0.25	0.01
TC	-0.33	**0.52***	-0.11	-0.31
TN	**-0.40***	**0.52***	-0.04	-0.20
C/N	-0.27	**0.49***	-0.13	-0.34
TP	0.38	-0.14	-0.03	-0.27
pH	0.18	-0.26	-0.13	0.25

Asterisks and bold print represent significant correlation (p < 0.05).

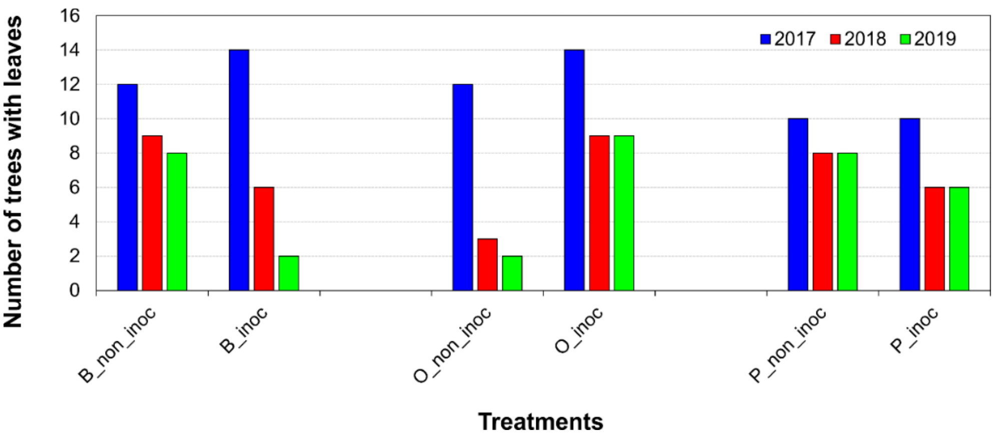

Figure S4. Plant growth during pot experiment. B – birch, O – oak, P – pine; non_inoc – non-inoculated variant of the experiment, inoc – inoculated variant of the experiment.

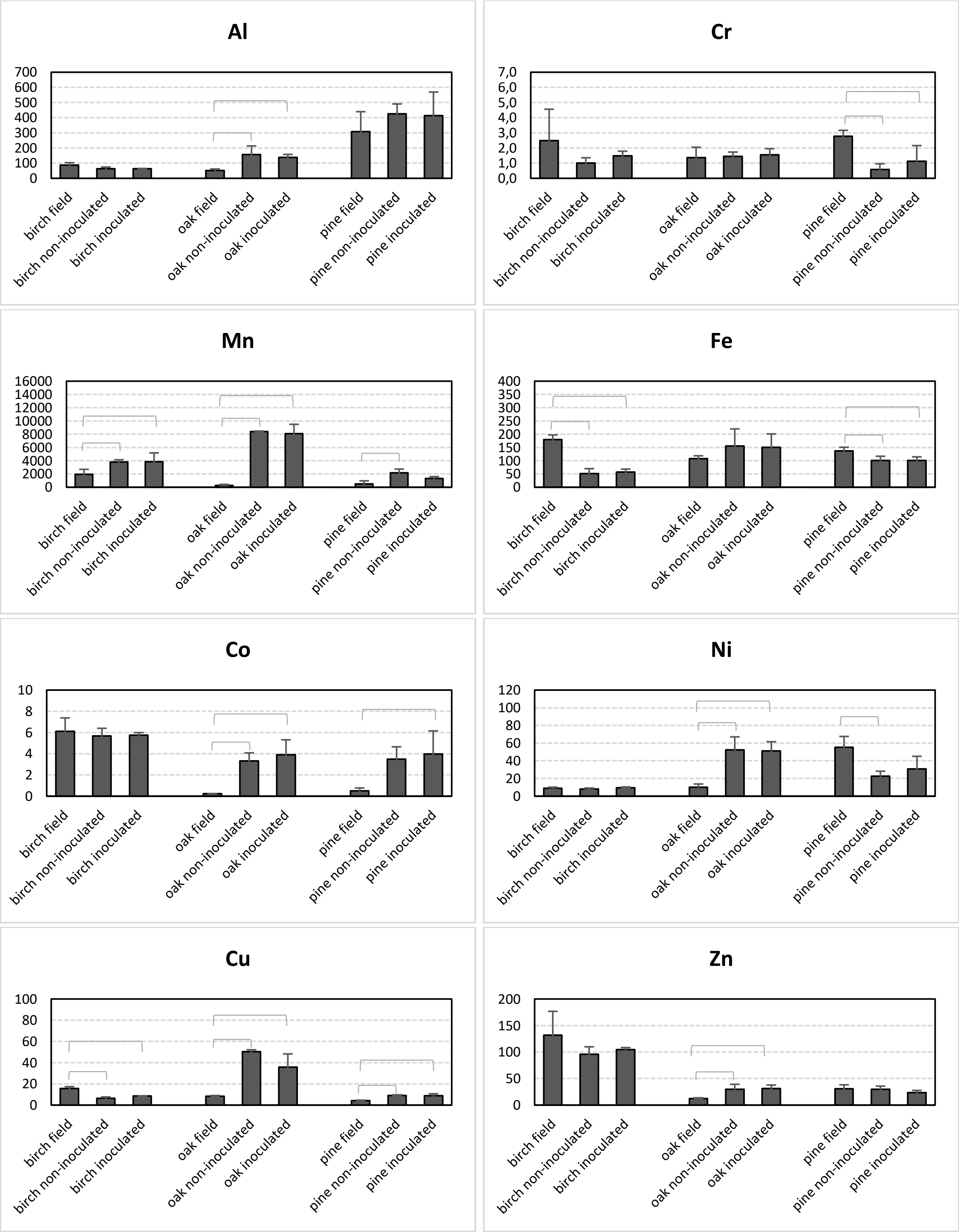
Al
0
100
200
300
400
500
600
700
Cr
0,0
1,0
2,0
3,0
4,0
5,0
6,0
7,0
Mn
0
2000
4000
6000
8000
10000
12000
14000
16000
Fe
0
50
100
150
200
250
300
350
400
Co
0
2
4
6
8
10
Ni
0
20
40
60
80
100
120
Cu
0
20
40
60
80
100
Zn
0
50
100
150
200
birch field
birch non-inoculated
birch inoculated
oak field
oak non-inoculated
oak inoculated
pine field
pine non-inoculated
pine inoculated

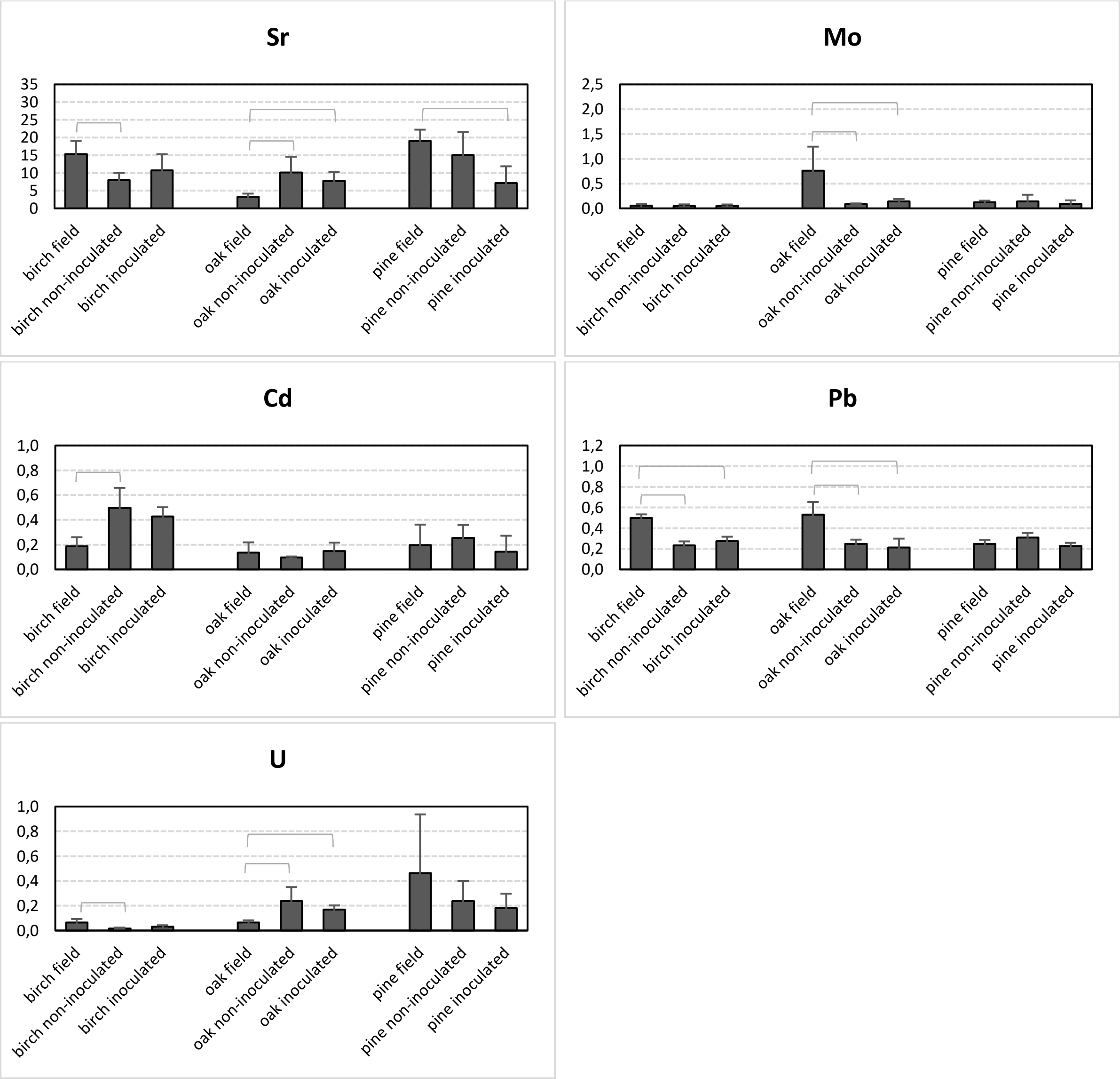

Figure S5. Content of toxic metals measured in aboveground dried plant biomass in variants of the experiment. y-axis represents concentration in µg/g. Brackets represent significant differences ($p < 0.05$).
